# Validation of minimal risk of recurrence classification by the Breast Cancer Index in early stage breast cancer

**DOI:** 10.1038/s41523-025-00885-x

**Published:** 2025-12-30

**Authors:** Marie-France Jilderda, John M. S. Bartlett, Gerrit-Jan Liefers, Yi Zhang, Hywel Dunn-Davies, Valerie Rebattu, Ranelle Salunga, Elma Meershoek-Klein Kranenbarg, Linda de Munck, Annette Hasenburg, Christos Markopoulos, Luc Dirix, Cornelis J. H. van de Velde, Daniel Rea, Amanda K. L. Anderson, Esther Bastiaannet, Kai Treuner, Karen J. Taylor

**Affiliations:** 1 Department of Surgery, Leiden University Medical Center, Leiden, the Netherlands; 2 Edinburgh Cancer Research, CRUK Scotland Centre, Institute of Genetics and Cancer, University of Edinburgh, Edinburgh, UK; 3 Biotheranostics Inc., a Hologic company, San Diego, CA USA; 4 MRC Human Genetics Unit, Institute of Genetics and Cancer, University of Edinburgh, Edinburgh, UK; 5 Department of Research and Development, Netherlands Comprehensive Cancer Organisation, Utrecht, The Netherlands; 6 Dept of Gynecology and Obstetrics, University Center Mainz, Mainz, Germany; 7 National and Kapodistrian University of Athens, Medical School, Athens, Greece; 8 St. Augustinus Hospital, Antwerp, Belgium; 9 Leiden University Medical Center, Leiden, the Netherlands; 10 Cancer Research UK Clinical Trials Unit, University of Birmingham, Birmingham, UK; 11 Epidemiology, Biostatistics and Prevention Institute, University of Zürich, Zurich, Switzerland

**Keywords:** Cancer, Medical research, Oncology

## Abstract

The Breast Cancer Index (BCI) was previously shown to identify ~20% of postmenopausal patients with early stage, hormone receptor positive (HR+), node negative (N0) breast cancer with minimal (<5%) risk of 10-year distant recurrence (DR) even without receiving adjuvant endocrine therapy (ET). This prospective-retrospective study further validated the BCI minimal risk classification in postmenopausal patients with early-stage, HR + HER2– N0 breast cancer from the Netherlands Cancer Registry (NCR) and the Tamoxifen and Exemestane Adjuvant Multinational (TEAM, NCT00279448, NCT00032136) randomized trial who received 5 years of primary adjuvant ET. BCI classified approximately 15% of patients as minimal risk. In the NCR cohort (*n* = 1264 out of 15,053 HR+ patients in the registry), risks of DR in the minimal, low, intermediate, and high groups were 4.8%, 3.3%, 8.0%, and 12.4%, respectively (*P* < 0.001). In the TEAM cohort (*n* = 978 out of 3544 in the BCI study), DR risks were 3.8%, 8.3%, 12.6% and 22.7% (*P* < 0.001). In multivariate analyses, BCI risk scores provided independent information over standard prognostic factors (*P* < 0.001). This study confirmed the ability of the adjusted BCI model to identify postmenopausal women with HR + HER2– N0 breast cancer who are at minimal risk of DR and may consider de-escalating adjuvant ET.

## Introduction

The standard of care treatment for hormone receptor–positive (HR+) breast cancer includes at least 5 years of adjuvant endocrine therapy (ET) with aromatase inhibitors and/or tamoxifen^1–5^, administered with the goal of reducing disease recurrence. However, the selection of the optimal therapy regimen and duration can be complex and requires consideration of the patient’s recurrence risk, comorbidities, and potential benefits and risks of adjuvant ET^1^. Accurate assessment of recurrence risk, to identify patients at very low risk of recurrence, can potentially spare many patients from overtreatment with adjuvant ET and the associated adverse effects such as musculoskeletal symptoms and bone toxicities with aromatase inhibitors and uterine cancer and deep vein thrombosis with tamoxifen^1,3–6^. Genomic biomarkers assessing the risk of recurrence may be useful to identify very low-risk patients at the time of diagnosis to guide treatment selection with the option of de-escalating adjuvant ET.

The Breast Cancer Index (BCI), a gene expression–based signature, reports both predictive^7–10^ and prognostic results^9–12^ to aid in clinical decision-making for extended adjuvant (post–year 5) ET in patients with early-stage, HR+ breast cancer. BCI predicts the likelihood of benefit from extended ET using the HOXB13/IL17BR expression ratio (H/I), an endocrine response biomarker that interrogates estrogen signaling in HR+ breast cancer^7,9,12,13^. BCI prognostic scores provide the individualized risk of overall (0–10 years after diagnosis) and late (>5 years after diagnosis) distant recurrence, calculated using an algorithm that combines BCI (H/I) and the Molecular Grade Index, which assesses tumor proliferative status based on the expression levels of 5 cell cycle–associated genes^10,12,14,15^. The prognostic component of BCI has been validated to predict the risk of overall and late distant recurrence in patients with HR + N0 breast cancer^12,14^. BCI is recommended in the National Comprehensive Cancer Network (NCCN) and American Society of Clinical Oncology (ASCO) clinical practice guidelines for clinical decision-making regarding extended ET^1,16^.

Recently, an additional BCI cutpoint was identified that stratified postmenopausal patients with early-stage, HR+, N0 breast cancer with minimal (<5%) risk of 10-year distant recurrence^17^. The study used patients from the Stockholm STO-3 trial and real-world data from the Netherlands Cancer Registry (NCR) that did not receive any adjuvant systemic therapy (ET or chemotherapy) to identify approximately 20% of patients in both cohorts with a minimal risk of 10-year distant recurrence. These patients classified as minimal risk are considered appropriate candidates for de-escalation of adjuvant ET.

Here we report further validation of the BCI minimal risk cutpoint in postmenopausal patients from the NCR and TEAM trial with early-stage, HER2–, N0 breast cancer who received primary adjuvant ET.

## Results

### Patient characteristics

Tumor specimens were collected from 1305 patients in the NCR cohort (Supplemental Fig. 1). After excluding 41 specimens that had insufficient tumor content or did not meet eligibility criteria, the final NCR dataset included 1264 patients who received adjuvant ET.

Table 1 summarizes clinicopathological characteristics of the NCR and TEAM cohorts. All patients in the NCR cohort were ≥70 years old, while most (63.8%) in the TEAM cohort were <70 years old. A higher proportion of patients in the NCR cohort had well-differentiated tumors compared with the TEAM cohort (21.5% vs 3.9%), and a lower proportion had poorly differentiated tumors (NCR, 17.6%; TEAM, 36.4%). The median follow-up in the NCR and TEAM cohorts was 12.2 and 9.4 years, respectively.

**Table 1:** Patient clinicopathologic characteristics in the NCR and TEAM cohorts

Characteristics	*n* (%)	
	NCR Cohort (*n* = 1264)	TEAM Cohort (*n* = 978)
Age at surgery, years		
Mean ± SD	78 ± 5	67 ± 8
<50	0	4 (0.4)
50–59	0	201 (20.6)
60–69	0	419 (42.8)
70–74	504 (39.9)	175 (17.9)
75–79	273 (21.6)	125 (12.8)
≥80	487 (38.5)	54 (5.5)
Tumor size, mm		
≤20	638 (50.5)	528 (54.0)
>20	626 (49.5)	449 (45.9)
Unknown	0	1 (0.1)
Tumor grade		
Well	272 (21.5)	38 (3.9)
Moderate	769 (60.8)	544 (55.6)
Poor	223 (17.6)	356 (36.4)
Unknown	0	40 (4.1)
PR status		
PR–	240 (19.0)	163 (16.7)
PR+	971 (76.8)	677 (69.2)
Unknown	53 (4.2)	138 (14.1)
Surgery type		
Mastectomy	703 (55.6)	401 (41.0)
Lumpectomy	561 (44.4)	577 (59.0)
Adjuvant endocrine therapy		
Tamoxifen	480 (38.0)	
Aromatase inhibitor	190 (15.0)	503 (51.4)
Sequential	415 (32.8)	475 (48.6)
Unknown	179 (14.2)	0
Disease recurrence		
Locoregional	26 (2.1)	26 (2.7)
Distant	83 (6.6)	93 (9.5)

*NCR* Netherlands Cancer Registry, *PR* progesterone receptor, *TEAM* Tamoxifen and Exemestane Adjuvant Multinational trial.

### Validation of the BCI minimal risk cutpoint in the NCR cohort

**Fig. 1:**
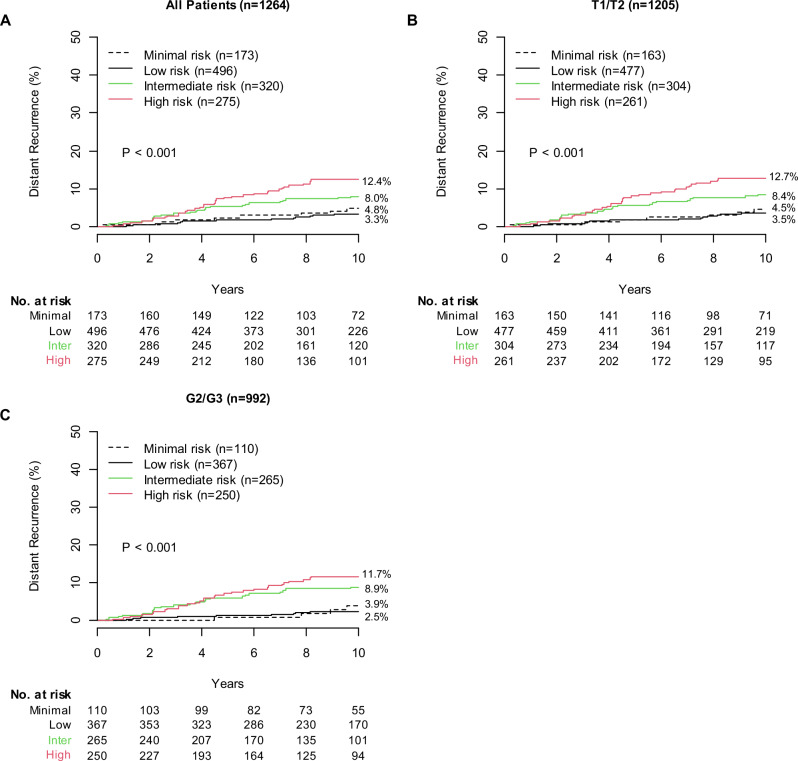
Performance of the adjusted BCI model in the NCR cohort. **A** NCR cohort overall. **B**, **C** Clinical subsets of the NCR cohort. BCI, Breast Cancer Index; G2/G3, tumor grade 2/3; NCR, Netherlands Cancer Registry; T1/T2, T stage 1/2; TAM, tamoxifen; TEAM, Tamoxifen and Exemestane Adjuvant Multinational trial.

The prognostic performance of the adjusted BCI model with the additional minimal risk cutpoint was evaluated first in patients from the NCR cohort (*n* = 1264), all of whom received up to 5 years of adjuvant ET. BCI classified 173 (13.7%), 496 (39.2%), 320 (25.3%), and 275 (21.8%) patients as minimal, low, intermediate, and high risk with 10-year risks of distant recurrence of 4.8% (95% CI, 2.3–8.9), 3.3% (95% CI, 2.0–5.2), 8.0% (95% CI, 5.3–11.4), and 12.4% (95% CI, 8.8–16.7), respectively (Fig. 1A, Table 2). The sHRs were 0.69 (95% CI, 0.30–1.60) for low risk, 1.73 (95% CI, 0.79–3.81) for intermediate risk, and 2.74 (95% CI, 1.28-5.87) for high risk versus minimal risk, with death as a competing risk event (*P* < .001) (Table 2).

**Table 2:** Prognostic performance of the four BCI risk groups

Cohort	Patients, no. (%)	10-year Distant Recurrence Risk % (95% CI)	(s)HR^a^ % (95% CI)	*P*
*NCR*	*n* = 1264			
BCI minimal risk	173 (13.7)	4.8 (2.3–8.9)	–	<0.001
BCI low risk	496 (39.2)	3.3 (2.0–5.2)	0.69 (0.30–1.60)	
BCI intermediate risk	320 (25.3)	8.0 (5.3–11.4)	1.73 (0.79–3.81)	
BCI high risk	275 (21.8)	12.4 (8.8–16.7)	2.74 (1.28–5.87)	
*TEAM*	n = 978			
BCI minimal risk	151 (15.4)	3.8 (0.4–7.0)	–	<0.001
BCI low risk	347 (35.5)	8.3 (4.1–12.3)	1.62 (0.60–4.37)	
BCI intermediate risk	280 (28.6)	12.6 (8.2–16.8)	3.36 (1.30–8.65)	
BCI high risk	200 (20.4)	22.7 (16.1–28.9)	6.51 (2.57–16.51)	

*BCI* Breast Cancer Index, *HR* hazard ratio, *NCR* Netherlands Cancer Registry, *sHR* subdistribution hazard ratio, *TEAM* Tamoxifen and Exemestane Adjuvant Multinational trial.

^a^HR was used for the TEAM cohort. sHR considering death as a competing risk was used for the NCR cohort.

The prognostic performance of the adjusted BCI model was further evaluated in relevant clinical subgroups. Among patients with T1/T2 disease (*n* = 1205), the 10-year risk of distant recurrence was 4.5% (95% CI, 2.0–8.7) for minimal risk, 3.5% (95% CI, 2.1–5.4) for low risk, 8.4% (95% CI, 5.6–11.9) for intermediate risk, and 12.7% (95% CI, 9.0–17.1) for high risk (Fig. 1B). The sHRs were 0.78 (95% CI, 0.32–1.87) for low risk, 1.98 (95% CI, 0.86–4.53) for intermediate risk, and 3.04 (95% CI, 1.36–6.81) for high risk versus minimal risk (*P* < .001).

Among patients with G2/G3 disease (*n* = 992), the 10-year risk of distant recurrence was 3.9% (95% CI, 1.3–9.1) for minimal risk, 2.5% (95% CI, 1.2–4.5) for low risk, 8.9% (95% CI, 5.8–12.7) for intermediate risk, and 11.7% (95% CI, 8.0–16.0) for high risk (Fig. 1C). The sHRs were 0.67 (95% CI, 0.21–2.16) for low risk, 2.50 (95% CI, 0.88–7.11) for intermediate risk, and 3.31 (95% CI, 1.19–9.25) for high risk versus minimal risk (*P* < 0.001).

To investigate whether BCI provided independent prognostic information over traditional clinicopatho-logical factors, univariate and multivariate Fine-Gray models were performed. Only BCI risk scores were significantly prognostic for 10-year distant recurrence in both univariate and multivariate analysis (Table 3), providing additional independent prognostic information over standard prognostic factors, with a sHR of 3.42 (1.87–6.26; likelihood ratio χ^2^ = 14.58; *P* < .001).

**Table 3:** Univariate and multivariate Fine-Gray regression analyses of BCI in the NCR cohort

Variable	Univariate Analysis		Multivariate Analysis	
	(s)HR (95% CI)	*P*	(s)HR (95% CI)	*P*
*NCR*				
Age^a^	0.73 (0.50–1.06)	0.10	0.65 (0.42–0.99)	0.05
Tumor size ( >20 mm vs ≤20 mm)	1.46 (0.95–2.26)	0.09	1.58 (0.95–2.65)	0.08
Tumor grade (G2/G3 vs G1)	0.99 (0.59–1.66)	0.98	0.78 (0.45–1.35)	0.37
Treatment (tamoxifen vs AI/sequential)	1.09 (0.69-1.71)	0.72	1.13 (0.72-1.81)	0.58
BCI^b^	3.63 (2.05–6.44)	<0.001	3.42 (1.87–6.26)	<0.001
*TEAM*				
Age^a^	1.33 (1.03–1.71)	0.03	1.30 (0.99–1.70)	0.05
Tumor size (>20 mm vs ≤20 mm)	1.91 (1.26–2.90)	0.003	1.66 (1.06–2.58)	0.03
Tumor grade (G2/G3 vs G1)	0.85 (0.31–2.33)	0.76	0.50 (0.17–1.42)	0.19
Treatment (tamoxifen vs exemestane)	0.99 (0.66–1.48)	0.94	1.08 (0.71–1.65)	0.72
BCI^b^	7.81 (4.30–14.18)	<0.001	7.49 (3.97–14.11)	<0.001

*BCI* Breast Cancer Index, *G1* tumor grade 1, *NCR* Netherlands Cancer Registry, *sHR* subdistribution hazard ratio, *AI* Aromatase Inhibitor, *TEAM* Tamoxifen and Exemestane Adjuvant Multinational trial.

^a^Age was analyzed in 10-year increments; in the NCR cohort, all patients were at least 70 years old. ^b^BCI was analyzed as a continuous variable with 5-unit increments.

### Validation of the BCI minimal risk cutpoint in the TEAM cohort

**Fig. 2:**
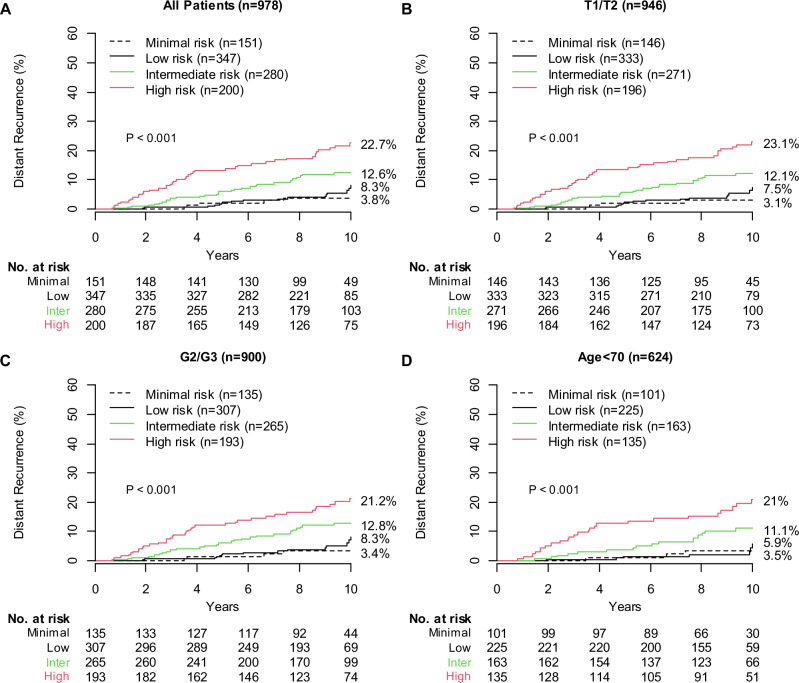
Performance of the adjusted BCI model in the TEAM cohort. **A** TEAM cohort overall. **B**–**D** Clinical subsets from the TEAM cohort. BCI, Breast Cancer Index; NCR, Netherlands Cancer Registry; T1/T2, T stage 1/2; G2/G3, grade 2/3; TEAM, Tamoxifen and Exemestane Adjuvant Multinational trial.

The prognostic performance of the adjusted BCI model was further validated in patients from the TEAM trial (*n* = 978). BCI categorized 151 (15.4%), 347 (35.5%), 280 (28.6%), and 200 (20.4%) patients as minimal, low, intermediate, and high risk, respectively (Fig. 2A, Table 2). The corresponding 10-year risks of distant recurrence were 3.8% (95% CI, 0.4–7.0), 8.3% (95% CI, 4.1–12.3), 12.6% (95% CI, 8.2–16.8), and 22.7% (95% CI, 16.1–28.9), respectively (Fig. 2A, Table 2). The HRs were 1.62 (95% CI, 0.60–4.37) for low risk, 3.36 (95% CI, 1.30–8.65) for intermediate risk, and 6.51 (95% CI, 2.57–16.51) for high risk versus minimal risk (*P* < 0.001) (Table 2).

Among patients with T1/T2 disease (n = 946), the 10-year risk of distant recurrence was 3.1% (95% CI, 0.0–6.0) for minimal risk, 7.5% (95% CI, 3.4–11.4) for low risk, 12.1% (95% CI, 7.7–16.3) for intermediate risk, and 23.1% (95% CI, 16.3–29.3) for high risk (Fig. 2B). The HRs were 1.81 (95% CI, 0.60–5.41) for low risk, 3.89 (95% CI, 1.36–11.08) for intermediate risk, and 7.98 (95% CI, 2.85–22.30) for high risk versus minimal risk (*P* < .001).

Among patients with G2/3 disease (*n* = 900), the 10-year risk of distant recurrence was 3.4% (95% CI, 0.0–6.6) for minimal risk, 8.3% (95% CI, 3.5–12.8) for low risk, 12.8% (95% CI, 8.3–17.1) for intermediate risk, and 21.2% (95% CI, 14.6–27.3) for high risk (Fig. 2C). The HRs were 1.75 (95% CI, 0.58–5.27) for low risk, 3.88 (95% CI, 1.36–11.03) for intermediate risk, and 6.80 (95% CI, 2.42–19.11) for high risk versus minimal risk (*P* < .001).

Among patients <70 years old (*n* = 624), the 10-year risk of distant recurrence was 3.5% (95% CI, 0.0–7.4) for minimal risk, 5.9% (95% CI, 1.0–10.6) for low risk, 11.1% (95% CI, 5.8–16.2) for intermediate risk, and 21.0% (95% CI, 13.1–28.2) for high risk (Fig. 2D). The HRs were 1.05 (95% CI, 0.27–4.04) for low risk, 3.16 (95% CI, 0.92–10.84) for intermediate risk, and 6.44 (95% CI, 1.94–21.34) for high risk versus minimal risk (*P* < .001).

In univariate and multivariate analyses, both tumor size and BCI risk scores were significantly prognostic for 10-year distant recurrence (Table 3). In multivariate analysis, the additional independent prognostic information that BCI risk scores provided over standard prognostic factors was associated with an HR of 7.49 (95% CI, 3.97–14.11; likelihood ratio χ^2^ = 41.31; *P* < 0.001).

## Discussion

Five years of adjuvant ET is part of the standard of care treatment for HR+ breast cancer, with the goal of reducing the risk of disease recurrence^1,2^. However, the rate of DR has declined in recent decades following improvements in adjuvant therapies and biomarker tests to better characterize the disease^18^. It is now understood that the risk of recurrence substantially varies among patients but can be predicted on an individual patient basis using clinicopathological risk factors and genomic classifiers^16,19,20^. Among patients with early-stage breast cancer, distant or multiple recurrences represent 71% of first recurrence events^21^. Many local recurrences are eventually followed by a DR event, which is associated with breast cancer–specific mortality^21,22^. DR is therefore the most clinically relevant endpoint when considering treatment decisions for early-stage breast cancer. Patients at minimal risk of distant recurrence may consider shortening ET treatment duration and avoiding the potentially serious toxicities associated with long-term adjuvant ET.

The prognostic ability of BCI to stratify patients with early-stage ER+ breast cancer based on the risk of overall (0–10 years) and late (5–10 years) DR has been demonstrated previously^12,14^. These initial studies classified patients according to low, intermediate, and high recurrence risk, allowing for the identification of patients most and least likely to benefit from adjuvant systemic therapy. Recently, an additional BCI cutpoint was developed and validated to identify a subgroup of postmenopausal patients with HR + N0 disease at minimal risk ( < 5%) of 10-year distant recurrence even without receiving any adjuvant ET^17^. In that study, which included patients from the prospective randomized Stockholm trial (untreated vs. 2 or 5 years of tamoxifen) and an untreated cohort from NCR, 16–22% of patients were classified as minimal risk of recurrence, with 10-year DR rates of 2.3–4.5% across cohorts. Notably, when analyzing patients in the two treatment arms (untreated vs. 2 or 5 years of tamoxifen) from the Stockholm trial, albeit the limited sample size, all patients categorized as low, intermediate and high risk derived substantial absolute benefit from adjuvant tamoxifen but not those in the minimal risk group. The reductions in risks of distant recurrence for minimal, low, intermediate and high risk patients were −2.0%, 10.5%, 8.1%, and 14.8%, respectively. As a result of the differential benefit across BCI risk groups, the minimal and low risk group displayed a similar risk profile during follow-up in the treated Stockholm cohort^17^.

The current analysis further validated the BCI minimal risk cutpoint in patients who received primary adjuvant ET. Similar to the previous validation^17^, approximately 15% of patients were identified as minimal risk (<5%) of 10-year DR. At year 10, 4.8% of minimal risk patients in the NCR cohort and 3.8% in the TEAM cohort had experienced a DR event. Notably, similar to what was observed in the Stockholm treated cohort, patients categorized as BCI minimal and low risk from both NCR and TEAM cohorts who were treated with adjuvant ET shared a similar risk profile across the 10 years of follow-up, suggesting that patients categorized as low risk may derive benefit from adjuvant ET, while those classified as minimal risk may not.

The immediate clinical utility of the minimal risk classification may mainly lie in patients deemed clinically low risk, as these patients are more likely to be considered for de-escalation of adjuvant ET. To that end, this study demonstrated that those patients with T1 or T2 tumors indeed experienced <5% risk of DR over 10 years (4.5% in the NCR cohort and 3.1% in the TEAM cohort) and thus could consider shorter duration of adjuvant ET to balance the potential benefit and harm of such treatment. Finally, since the NCR cohorts only included patients who were at least 70 years old, the subset analysis of patients from the TEAM trial who were younger than 70 years confirmed the prognostic performance of the minimal risk group in postmenopausal women regardless of age.

Study strengths include the use of a large, prospectively collected real-world patient cohort and data from a large, randomized controlled trial. In the NCR cohort, the estimated 10-year risk of distant recurrence rates are based on the assumption of having completed 5 years of ET, however the actual duration of ET may have been shorter, therefore the rates could be an overestimation of the true 10-year risk of DR. One limitation was the exclusion of patients ≤70 years old from the NCR cohort; however this was balanced by including the TEAM cohort with 64% of patients <70 years old. A high mortality rate in the NCR cohort (79%; not breast cancer–specific) during the 10-year follow-up posed another limitation to the study. This was addressed by considering death as a competing risk event in the analyses but might still lead to an underestimation of recurrence risk, especially in patients categorized as high risk. Additional studies may further elucidate the prognostic ability of the BCI minimal risk cutpoint beyond 10 years and in premenopausal patients who face the challenging clinical decision of whether to add ovarian function suppression to adjuvant ET.

In conclusion, the results of this study further validate BCI as a biomarker for personalized adjuvant ET decision-making to avoid over- and undertreatment of patients with early-stage, HR+ breast cancer. The use of BCI at the time of diagnosis or within the first 5 years can inform the risk of distant recurrence and, for patients with minimal risk of recurrence, may support decisions to consider de-escalating adjuvant ET.

## Methods

### Study design and patient selection

The primary study objective was to examine whether the adjusted BCI risk model would identify a group of women with HR + HER2– N0 breast cancer who received adjuvant ET and were at minimal risk (<5%) of 10-year distant recurrence. Secondary objectives were to evaluate the prognostic performance of the adjusted BCI model in patient subgroups according to tumor size, grade and age. The study endpoint was time to distant recurrence, defined as time from randomization (TEAM trial) or date of diagnosis (NCR cohort) to first recurrence at distant sites. Contralateral disease, locoregional recurrence, and other secondary primary cancers were not counted as events.

The phase 3 randomized TEAM trial evaluated disease-free survival in postmenopausal women with HR+ breast cancer who received 5 years of adjuvant treatment with exemestane alone or tamoxifen for 2.5-3 years followed by exemestane (NCT00279448, NCT00032136, and NCT00036270)^23^. The previous BCI translational study evaluated 3544 patients from the TEAM trial who had available RNA samples for BCI testing^24^. The current analysis included patients with HER2− N0 breast cancer who received adjuvant ET but no chemotherapy. The cutoff level for ER and PR positivity was set to 1% as previously described^24^.

The NCR cohort included patients ≥70 years old with a diagnosis of invasive, ER + HER2-, N0 breast cancer who had undergone surgery and received adjuvant ET but no chemotherapy from 45 hospitals throughout the Netherlands^25^. Specially trained data managers from NCR collected clinical data on diagnosis, staging, and treatment directly from medical records using international coding rules. In compliance with Dutch national breast cancer pathology guidelines, the cutoff level for ER positivity was set to 10%^26^. Information on disease recurrence was retrospectively collected from electronic medical records. Registration and distribution of formalin-fixed, paraffin-embedded tumor blocks acquired during routine treatment were coordinated by Palga, the nationwide network and registry of histo- and cytopathology in the Netherlands.

The TEAM trial and NCR study were conducted in accordance with the Declaration of Helsinki and the International Council for Harmonisation guidelines, as well as Good Clinical Practice. The collection and analysis of tumor samples from the TEAM trial were approved by the institutional review boards, and all patients provided written informed consent. The TEAM trial is registered with ClinicalTrials.gov, NCT00279448 and NCT00032136. The NCR study was ethically approved by the Institutional Review Board of the Leiden University Medical Center. Due to the large number of deceased participants, the study was granted a waiver for informed consent.

### BCI cutpoints and molecular testing

The traditional BCI prognostic model stratifies patients into low, intermediate, and high risk groups using validated cutpoints of 5.1 and 6.5^12^. Recently, an additional minimal risk cutpoint of 3.0 was developed and validated to identify patients who had less than a 5% risk of 10-year distant recurrence even without receiving adjuvant ET^17^. These cutpoints would stratify patients into four prognostic risk groups (minimal, low, intermediate, high risk). In the current study, this adjusted BCI model was further validated using patients in the TEAM trial and the NCR cohort who received adjuvant ET.

The BCI analysis of the TEAM cohort was described previously^24^. For the NCR cohort, analysis of BCI gene expression was done using RT-qPCR with RNA extracted from primary tumor specimens. Tumor sections were prepared at Leiden University Medical Center, shipped to Biotheranostics Inc., a Hologic company, and tested in the CLIA-certified, CAP-accredited laboratory. Testing was blinded to clinical outcome and done in accordance with Good Clinical Practice. Total extracted RNA was reverse transcribed. The resulting cDNA was pre-amplified using PCR with the PreAmp Master Mix Kit (Thermo Fisher Scientific; Carlsbad, CA) and was analyzed using TaqMan RT-PCR. The final analysis included all samples that met predetermined quality control criteria requiring a mean cycle threshold of 4 reference genes of <26.5^12^. BCI scores were calculated and patients were stratified into the four prognostic risk groups as described above.

### Statistical considerations

In the TEAM cohort, Kaplan-Meier survival analysis was used to graphically present survival curves for the adjusted BCI risk groups; equality of the survival curves was evaluated using a log-rank test. Cox proportional hazards models were used to calculate hazard ratios (HRs) and 95% CIs. In the NCR cohort, to account for the death of 79% of subjects, cumulative incidence analysis and sub-distribution HRs (sHRs) from Fine-Gray models with likelihood ratio tests were used, both analyses considering death as a competing risk event. For both cohorts, univariate and multivariate Cox or Fine-Gray models with likelihood ratio tests were used to evaluate whether BCI as a continuous risk index (analyzed in 5-unit increments) provided additional prognostic information independent of clinical and pathologic factors. For all analyses, follow-up was truncated at 10 years. A two-sided *P* ≤ .05 was considered statistically significant. Statistical analyses were done using R (version 4.3.1).

## Supplementary information

Supplementary Information

## Data Availability

Qualified researchers may request access to de-identified individual participant data by submitting a proposal to the corresponding author, which will be reviewed for scientific merit and feasibility.
